# Role of 18F-PET-CT to predict pathological response after neoadjuvant treatment of rectal cancer

**DOI:** 10.1007/s12672-021-00405-w

**Published:** 2021-05-18

**Authors:** Riccardo Caruso, Emilio Vicente, Yolanda Quijano, Hipolito Duran, Isabel Fabra, Eduardo Diaz, Luis Malave, Ruben Agresott, Lina García Cañamaque, Benedetto Ielpo, Valentina Ferri

**Affiliations:** 1 General Surgery Department, Sanchinarro University Hospital, San Pablo University, CEU, C/Oña No. 10, 28050 Madrid, Spain; 2 Division of Nuclear Medicine, Sanchinarro Hospital, San Pablo University, Madrid, Spain; 3 HPB Unit, University Parc Salut Mar Hospital, Barcelona, Spain

## Abstract

**Objectives:**

Neoadjuvant chemoradiation (nCRT) is universally considered to be a valid treatment to achieve downstaging, to improve local disease control and to obtain better resectability in locally advanced rectal cancer (LARC). The aim of this study is to correlate the change in the tumour 18F-FDG PET-CT standardized uptake value (SUV) before and after nCRT, in order to obtain an early prediction of the pathologic response (pR) achieved in patients with LARC.

**Data description:**

We performed a retrospective analysis of patients with LARC diagnosis who underwent curative resection. All patients underwent a baseline 18F-FDG PET-CT scan within the week prior to the initiation of the treatment (PET-CT SUV1) and a second scan (PET-CT SUV2) within 6 weeks of the completion of nCRT. We evaluated the prognostic value of 18F-FDG PET-CT in terms of disease-free survival (DFS) and overall survival (OS) in patients with LARC.A total of 133 patients with LARC were included in the study. Patients were divided in two groups according to the TRG (tumour regression grade): 107 (80%) as the responders group (TRG0-TRG1) and 26 (25%) as the no-responders group (TRG2-TRG3). We obtained a significant difference in Δ%SUV between the two different groups; responders versus no-responders (p < 0.012). The results of this analysis show that 18F-FDG PET-CT may be an indicator to evaluate the pR to nCRT in patients with LARC. The decrease in 18F-FDG PET-CT uptake in the primary tumour may offer important information in order for an early identification of those patients more likely to obtain a pCR to nCRT and to predict those who are unlikely to significantly regress.

## Introduction

Nowadays neoadjuvant chemoradiation (nCRT) is universally considered to be a valid treatment to achieve downstaging, to improve local disease control and to obtain better resectability in locally advanced rectal cancer (LARC) [1]. Currently, about 15–30% of patients undergoing neoadjuvant treatment achieve a pathologic complete response (pCR) with improved oncological outcomes [2]. A major challenge for the surgeon is to determine the course of treatment for those patients with pCR after nCRT [3–5]. In fact, the difficult choice between surgery (radical rectal resection or trans-anal local excision of the residual scar) and the ‘wait-and-see’ strategy does not provide a definitive or widely-applicable answer. The pCR is defined through endoscopy with biopsy and through radiological studies. However, a more reliable histopathologic response is obtained only after the resected specimen analysis [6, 7]. For this reason, it is important to develop methods to identify an early prediction of histopathological response in patients with LARC after nCRT. The early determination of pathological response to nCRT is important not only for prognostic value but also to change or adapt the standard nCRT strategy for those patients with a suboptimal pathologic response [8]. At this time, 18F-FDG PET-CT is one of the most powerful tools in cancer diagnosis and staging. It combines a positron emission tomography scanner (PET) and an X-ray computed tomography (CT) scanner, so that images acquired from both devices can be taken sequentially. The spatial distribution of metabolic or biochemical activity in the body can be more precisely aligned or correlated with anatomic imaging obtained by CT scanning. It is well known that nCRT induces changes in tumour metabolism that influence the 18F-FDG PET-CT tumour uptake [9].

## Materials and methods

### Patient population

We performed a retrospective analysis of patients with LARC diagnosis who underwent curative resection. The study was carried out at the General Surgery Department of the Sanchinarro University Hospital, Madrid, recruiting patients between October 2011 and February 2020 with LARC. The LARC patients are defined using radiological staging T3-4 e/o N + rectal tumour with a distance from the anal verge ≤ 10 cm. Patients who are younger than 18 years old, with concomitant metastases, perforated tumours, peritoneal carcinomatosis or comorbidities precluding surgery and neoadjuvant therapy with rectal stent, are excluded from the analysis. All included patients received nCRT, and surgical treatment was carried out 8–12 weeks after completing the neoadjuvant treatment. The preoperative study included a colonoscopy with biopsy to confirm rectal adenocarcinoma. Trans-anal endorectal ultrasound, pelvic MRI (magnetic resonance imaging), total body CT scan, and PET-CT scan were used as diagnostic and staging procedures. For the tumoral stage, the TNM staging system was used (American Joint Committee on Cancer) [10].

Pathologic tumour staging of the specimen after nCRT (ypTNM) and tumour regression grade (TRG) scores of the surgical specimens were established using the Seventh Guidelines of the American Joint Committee on Cancer [11]. TRG0 indicates a pathological complete response, TRG1 (moderate response) consists of single cells or small groups of cancer cells, TRG2 (minimal response) indicates residual cancer outgrown by fibrosis, and TRG3 (poor response) shows extensive residual cancer. Circumferential resection margin (CRM) is considered negative if the distance between the tumour and CRM is more than 1 mm [6]. Patients were divided into two groups according to the TRG: the responders group (TRG0-TRG1) and the no-responders group (TRG2-TRG3). Furthermore, we evaluated the prognostic value of 18F-FDG PET-CT in terms of disease-free survival (DFS) and overall survival (OS) in patients with LARC. Following nCRT, patients were reassessed to evaluate the tumour’s response 6 weeks after completing the treatment. The re-evaluation study included a full physical examination, MRI, CT-scan, and PET-CT scan. All resected specimens were examined by an experienced team of gastrointestinal pathologists. We excluded patients with stage IV and patients who were lost during follow up.

### Preoperative chemoradiation (nCRT)

All patients were treated with preoperative intensity-modulated radiotherapy (IMRT) and an integrated-boost chemoradiation scheme. The planning target volume (PTV) included the presacral node, the tumour, the complete mesorectal fascia, and common and internal iliac lymph nodes. Radiation therapy was completed within 4–5 weeks, with a total of 23 fractions. The dose for the first PTV was 46 Gy in 23 fractions, and the concomitant boost (PTV2) was 57.5 Gy in 23 fractions (BED = 71.8 Gy[α/β = 10] and Eq. 2 Gy/f = 70 Gy). All patients received concurrent standard capecitabine-based chemotherapy (825 mg/m^2^, in bid) and a blood count was performed every 14 days. None of the patients included in the study received a short-course of CRT. All patients underwent a surgical procedure, including a low anterior resection or abdominoperineal excision 6–8 weeks after the completion of treatment. All patients were operated on by the same team of surgeons and with mechanical bowel preparation. Our study adhered to the STROBE recommendation for observational studies.

### 18F-FDG PET-CT analysis

All patients underwent a baseline 18F-FDG PET-CT scan within the week prior to the initiation of the nCRT (PET-CT SUV1) and a second scan (PET-C T SUV2) within 6 weeks of the completion of nCRT. The 18F-FDG PET-CT was performed with the advance whole-body PET scanner in 3D mode, with axial spatial resolution of 4.7 mm. CT-based attenuation and decay correction were done. Patients were fasting six hours before they underwent PET-CT, although water intake was encouraged. Before entering the scanner, patients were invited to drink water (1 l) and then void their bladder. Fasting serum glucose levels were checked 15 min before the FDG injection according to protocol. All patients received an intravenous injection of 18F-FDG. The exact time of injection of 10–15 mCi of 18F-FDG was recorded and imaging commenced no earlier than 45 min after the injection. Total body, caudo-cranial 18F-FDG PET-CT images were acquired 70 min after the injection of18F-FDG. PET-CT images were reconstructed from the acquired data, using the ordered subset expectation maximum iterative reconstruction algorithm. The maximum uptake value of the primary tumour was registered in all studies (baseline and after n weeks). SUVmax and SUVmean were calculated using the maximum and mean activity values with the highest radioactivity concentration in accordance with the injected dose and patient’s body weight. Changes in SUVmax values were analysed as the percentage difference from 18F-FDG PET-CT 1 (before nCRT) and 18F-FDG PET-CT 2 (after nCRT) (Δ% SUV = ($\frac{SUVpre-SUVpost}{\mathrm{SUVpre}}100\%$)). It was evaluated in relation to the pathologic response, defined as the TRG [6].

### Statistical analysis

Sensitivity, specificity, accuracy, positive predictive value (p-PV), and negative predictive value (n-PV) of post nCRT 18F-FDG PET-CT were assessed. To compare the correlation between the quantitative (numerical) variables, when these followed a normal distribution, a variance analysis and a t-Student were used. Categorical variables were assessed using a Chi-square test. The Mann–Whitney U-Test and the Kruskal–Wallis test were performed for numerical variables. A receiver-operating characteristic (ROC) curve and logistic regression techniques were used to obtain the predictive model and the inflection point. DFS and OS were calculated with the Kaplan–Meier method and log-rank test. A multivariate survival analysis for disease-free survival or overall survival was performed using the Cox proportional-hazard regression model. All variables related to the risk of DFS, or OS with a P value of < 0.2 in univariate analysis, were included in the multivariate analysis. For the statistical analysis, SPSS software (version 10, IBM SPSS, Chicago, IL, USA) was used and all tests were considered statistically significant if the value of p ≤ 0.05.

#### Ethics

The study was approved by the institutional ethical committee of Sanchinarro University Hospital, Madrid, and all patients included were informed about the treatment and provided written informed consent. The study was conducted in agreement with the Declaration of Helsinki for studies in humans.

#### Study aims

The aim of this study is to correlate the change in tumour 18F-FDG PET-CT standardized uptake value (SUV) before and after nCRT in patients with LARC, in order to differentiate between those who responded to treatment (the responders) and those who did not respond (the no-responders).

## Results

**Fig. 1:**
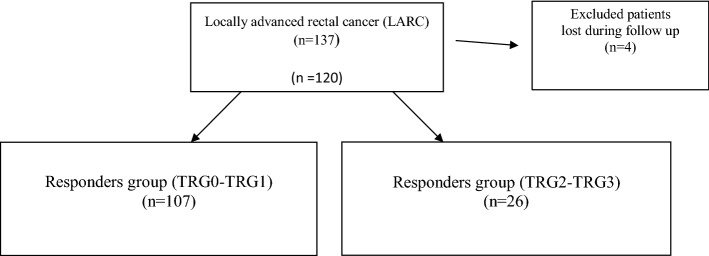
Flowchart

**Fig. 2:**
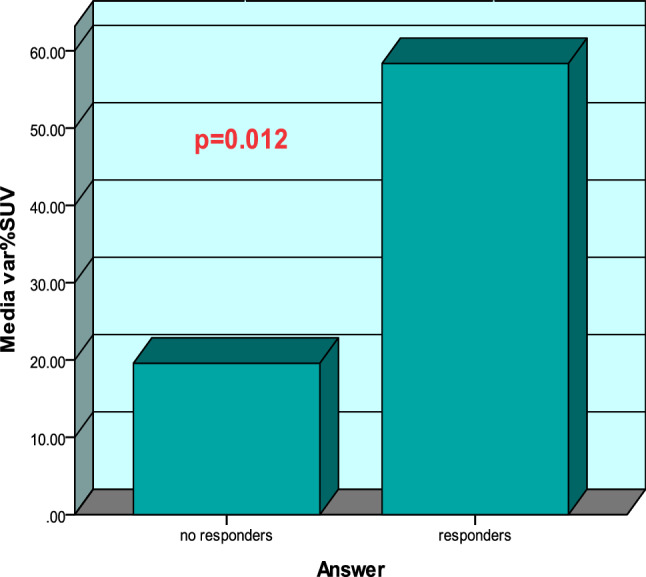
Difference in Δ%SUV between the responders and no responders group

**Fig. 3:**
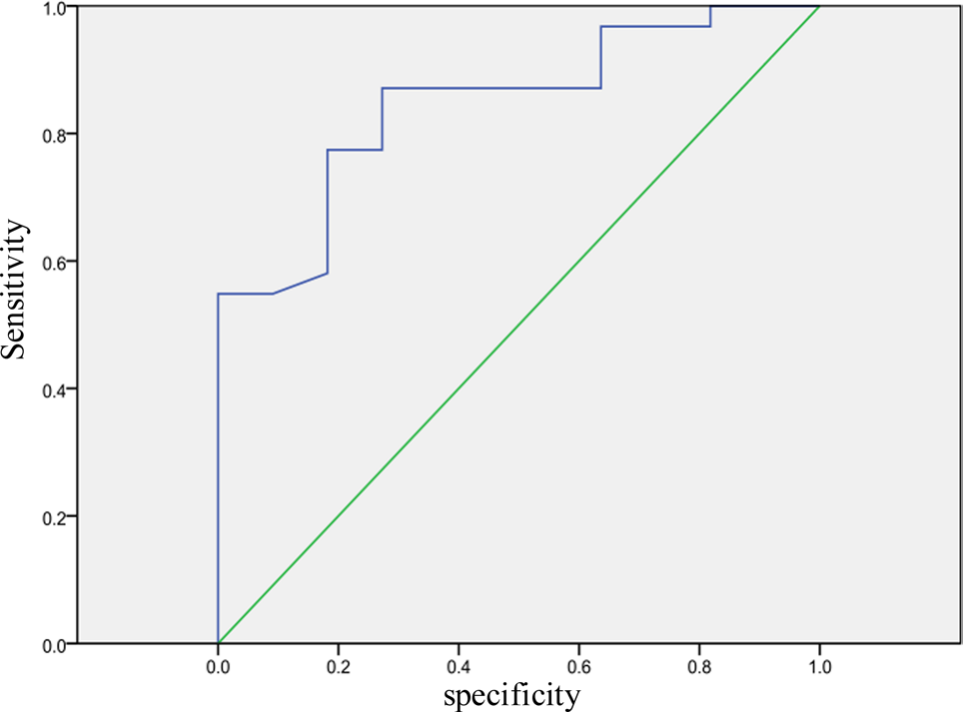
ROC (receiver operating characteristic) curve

A total of 137 patients with LARC were included and four were lost during follow-up as they underwent surgery in another centre and data could not be gathered. Therefore, 133 cases have been analysed (STROBE flowchart Fig. 1). The demographic data of the patients are summarized in Table 1. A total of 29 (22%) patients underwent abdominoperineal excision of the rectum (APER), 90 (68%) underwent a low anterior resection (LAR) with protective stoma and 14 (10%) underwent a LAR without protective stoma, as shown in Table 2. Patients were divided in two groups according to the TRG: 107 (80%) for the responders group (TRG0-TRG1) and 26 (25%) the no-responders group (TRG2-TRG3) (STROBE flowchart Fig. 1). Evaluation of the tumour response to nCRT according to TRG score is shown in Table 3. We found a primary tumour downstaging in T classification after nCRT in 85 patients (65%). Therefore, nodal downstaging after nCRT was achieved in 55 patients (42%). In our series the pathological complete response (cpR) rate was about 19%. A logistic regression analysis was performed in order to obtain a prediction model of the tumour’s pathological response (pR). Clinical prognostic factors (age, sex, TNM variables, tumoral markers, and FDG-PET values) were separately tested. From our statistical analysis, we obtained a significant difference in Δ%SUV between the two groups, the responders (Δ% SUV = 58%) versus the no-responders (Δ% SUV = 19%) (p < 0.012), as shown in Fig. 2. ROC curve preliminary cut-off value of 70% of the was individuate, as depicted in Fig. 3. This showed that Δ%SUV media was a stronger discriminator between the two groups with a high accuracy of 81% (34/42), a sensitivity of 84.4%, a specificity of 80%, a positive predictive value of 81.4% (p-PV), and a negative predictive value of 84.2% (n-PV).

**Table 1:** Patients and tumor characteristics

Median age (range)	62.8 (33–79)
Sex	
Male	70 (53%)
Female	63 (47%)
ASA	
I	19 (14%)
II	76 (57%)
III	38 (29%)
Clinical T stage	
cT3	100 (75%)
cT4	33 (25%)
Clinical N stage	
cN0	47 (36%)
cN1	67 (50%)
cN2	19 (14%)
Location of the tumor	
Upper third	21 (16%)
Middle third	77 (58%)
Lower third	35 (26%)

**Table 2:** Operative data

Type of resection	
APER	20 (15%)
LAR	19 (14%)
LAR with stoma	94 (70%)
Mean operative time (min) (SD)	280 ± 38
Intraoperative blood (ml) (SD)	205 ± 26
Hospital stay (days) (SD)	12.42 ± 7.77

*APER* abdominoperineal excision of the rectum, *LAR* low anterior resection, *SD* standard deviation

**Table 3:** Evaluation of the tumor response (TRG score)

	TRG				
	TRG 3	TRG 2	TRG 1	TRG 0	Total
Responders 107(80%)	0 (0)	0 (0)	75 (56)	32 (24)	107 (80)
No responders 26(25%)	22 (17)	4 (8)	0 (0)	(0)	26 (25)

*TRG* tumor regression grading

The median follow-up period was 60.54 months (a range of 9–103 months). Patients with overall recurrence totalled 25 (18.8%) (three locoregional and 22 metastatic): 17 (12.8%) patients with delta Δ% SUV < 70% and 8 (6%) patients with Δ% SUV > 70% with a significant correlation between recurrence and Δ SUV (p = 0.037).

**Fig. 4:**
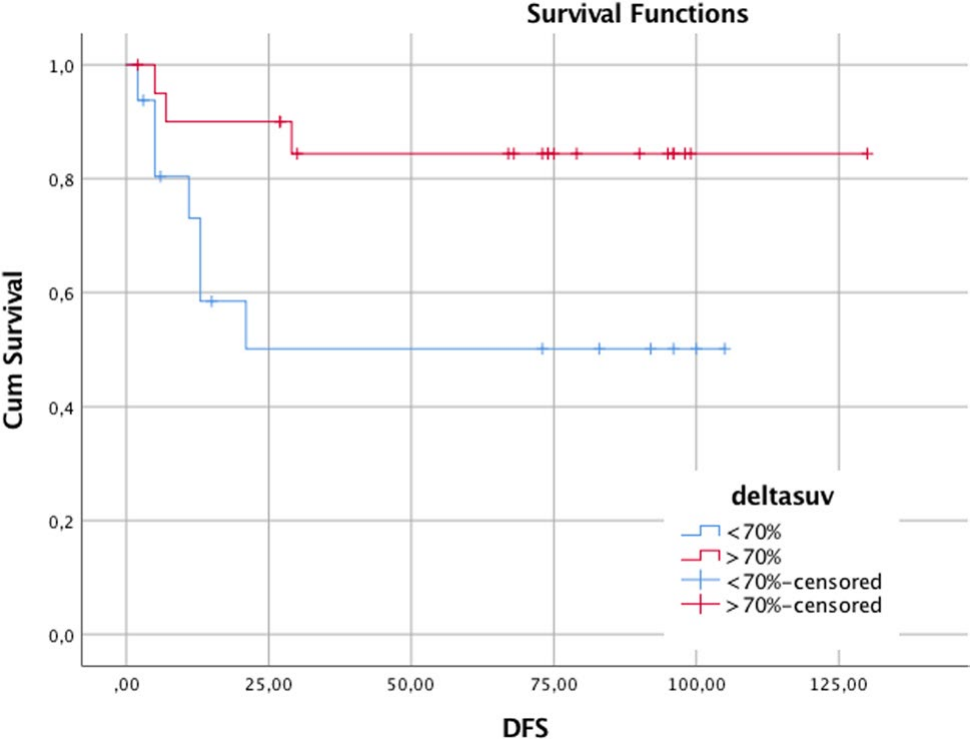
DFS (disease free survival) curve for the Δ SUV evaluation

The median 5-years DFS was 55.3 months (a range of 9–92 months). In the group of patients with delta SUV < 70% DFS was 56.3% mean, while in patients with delta SUV > 70% DFS was 85.7%, showing a statistical significance (p < 0.05) (Fig. 4).

In the multivariate analysis, ΔSUV results an independent risk factors, associated with local recurrence-free survival, as well as pTN stage and preoperative ASA (Table 4).

**Table 4:** Univariate and multivariate analysis DFS (disease free survival)

Variable	Univariate analysis		Multivariate analysis	
	Hazard ratio	p values	Hazard ratio	p values
Age (years, SD)				
< 60 > 60	1 2.509 (0.531–11.855)	0.224		
Sex				
F M	1 0.850 (0.246–2.941)	0.795		
ASA				
I–II III	1 3.328 (0.931–11.892)	0.064	1 5.074 (1.086–23.710)	0.39
CEA				
> 5 < 5	1 1.706 (0.340–4.067)	0.724		
Tumor localization				
> 10 cm < 10 cm	1 1.007 (0.278–14.172)	0.914		
Approch				
Laparoscopic Robotic	1 2.297 (0.487–10.830)	0.293		
pTNM				
0–I II–II	1 3.717 (0.786–17.569)	0.098	1 11.088 (1.451–84.720)	0.20
TRG				
0–1 2–3	1 2.801 (0.537–8.056)	0.268		
Lymphonodal ratio				
0 < 0.24 > 0.25	1 0.737 (0.89–6.097) 0.717 (0.64–8.064)	0.961		
Blood trasfusion				
No Yes	1 3.120 (0.866–11.239)	0.821	1 3.830 (0.866–16.932)	0.077
Dindo-Clavien ≥ 3				
No Yes	1 1.594 (0.409–6.211)	0.542		
SUV pre				
< 8 > 8	1 1.806 (0.222–14.702)	0.572		
SUV post				
< 8 > 8	1 2.090 (0.535–8.166)	0.271		
Δ SUV				
< 70% > 70%	1 4.078 (1.046–15.900)	0.043	1 4.793 (1.019–22.553)	0.047
CM affected				
Yes No	1 1.598 (0.632–5.317)	0.954		

*CM* circumferencial margin

**Fig. 5:**
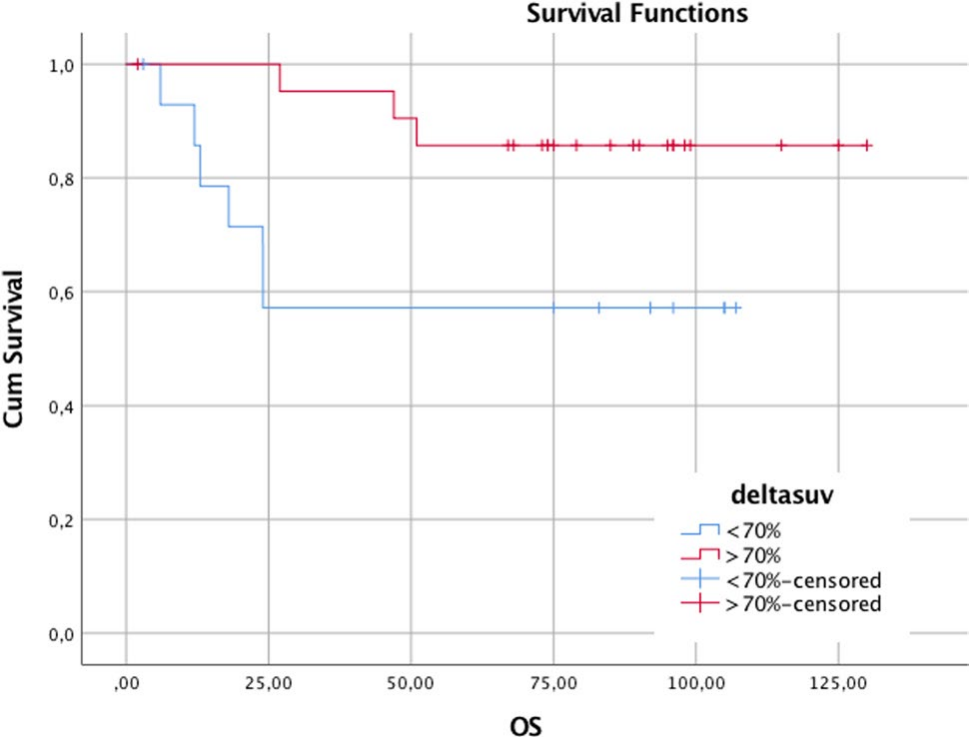
OS (overall survival) curve for the ΔSUV evaluation

The 5-years OS (overall survival) rate result was 60% in patients Δ% SUV < 70% and 86.4% in patients Δ% SUV > 70%, showing a statistical significance (p < 0.05) (see Fig. 5). The unadjusted Cox-proportional hazards regression revealed that delta SUV and pTN were associated with worse overall survival (Table 5).

**Table 5:** Univariate and multivariate analysis OS (overall survival)

Variable	Univariate analysis		Multivariate analysis	
	Hazard ratio	p values	Hazard ratio	p values
Age (years, SD)				
< 60 > 60	1 2.464 (0.511–11.878)	0.261		
Sex				
F M	1 1.472 (0.395–5.486)	0.565		
ASA				
I–II III	1 2.611 (0.652–10.448)	0.175	1 4.851 (0.752–21.283)	0.097
CEA				
> 5 < 5	1 1.102 (0.296–4.107)	0.908		
Tumor localization				
> 10 cm < 10 cm	1 1.133 (0.283–4.533)	0.860		
Approch				
Laparoscopic Robotic	1 4.921 (0.615–39.385)	0.133	1 5.217/0.624–43.650)	0.127
pTNM				
0–I II–II	1 8.158 (1.017–64.437)	0.048	1 15.315 (1.550–151.339)	0.20
TRG				
0–1 2–3	1 2.126 (0.531–8.504)	0.286		
Lymphoonodal ratio				
0 < 0.24 > 0.25	1 0.606 (0.73–5.042) 0.634 (0.57–7.000)	0,898		
Blood trasfusion				
No Yes	1 2.065 (0.515–8.280)	0.306		
Dindo-Clavien > 3				
No Yes	1 1.093 (0.227–5.268)	0.911		
SUV pre				
< 8 > 8	1 1.728 (0.212–14.056)	0.609		
SUV post				
< 8 > 8	1 2.514 (0.514–10.086)	0.271		
Δ SUV				
< 70% > 70%	1 4.060 (1.010–16.319)	0.048	1 7.629 (1.174–49.591)	0.033
CM affected				
Yes No	1 1.362 (0.532–5.435)	0.834		

*CM* circumferencial margin

The datasets generated during and/or analysed during the current study are available from the corresponding author on reasonable request.

## Discussion

Radical surgery with TME (total mesorectal excision) remains the main curative treatment for patients affected by LARC [12]. The TME associated with nCRT improve outcomes by increasing the 5-year survival rate [9]. Several previous studies have demonstrated that, compared with adjuvant treatment, preoperative nCRT significantly improves loco-regional tumour recurrence and reduces toxicity compared with postoperative strategies [10]. The early identification of pR after nCRT in rectal cancer remains an important challenge, and could avoid surgical overtreatment without compromising local control and long-term survival [12]. It could considerably reduce the number of surgical procedures required in the future, allowing less invasive procedures, such as TAMIS (trans-anal minimally invasive surgery), to be initially performed for LARC [3]. This latter approach could also reduce the rates of mortality, morbidity and other unsatisfactory functional outcomes that may occur after rectal resection. Furthermore, an early prediction in the no-responders group could provide the clinician the opportunity to evaluate the possibility of the reorientation of the standard treatment by also increasing the number of chemotherapy cycles and radiotherapy. The current conventional radiology, such as endorectal ultrasound (EU), CT scan and MRI for monitoring the tumour response, shows several limitations to assess the pR after nCRT. This is mainly due to the difficulties in discerning between disease persistence and radiation induced inflammation and fibrosis after nCRT [13]. 18F-FDG PET-CT has a recognized validity for monitoring nCRT effects [14–18].

There are several studies that have investigated the predictive value of 18F-FDG PET-CT in the LARC and in recurrent rectal cancer, and additional studies are likely to be conducted using collaborative, international research platforms [18–27]. However, these studies have some limits, mainly due to the methodological heterogeneity secondary to their multicentric nature in terms of preoperative studies, chemoradiotherapy, patients’ characteristics and the PET study method (timing, technique and analysis of images). Despite these limits, it is important to note that almost all of these previous studies identify a significant correlation between tumour 18F-FDG uptake and pR, and a correlation between OS and DFS [13].

A recent study by Niccoli-Asabella et al. was able to evaluate the prognostic value of 18F-FDG PET-CT in terms of survival in patients with LARC who have undergone surgery after nCRT. This work showed a high percentage of patients with a TRG complete response (39.7%) with longer OS and DFS in the Responders group but without statistically significant differences [13].

The strength of our study relies on being one of the largest studies evaluating 18F-FDG PET-CT related to histopathological response (TRG score) at two time-points: before nCRT (early PET-CT) and after finishing CRT (late PET/CT). We found the optimal cut-off to distinguish the responders (TRG3-TRG4) from the no-responders (TRG0-TRG2), at 70% of the Δ%SUV. Furthermore, our analysis showed that Δ%SUV was a stronger discriminator between the two groups with a high accuracy of 81% (34/42), with a sensitivity of 84.4%, a specificity of 80%, a positive predictive value of 81.4%, and a negative predictive value of 84.2%. We were able to find a correlation between Δ%SUV and OS and DFS, also showing a statistical significance (p < 0.05).

Another similar study by Leccisotti et al. found similar results They learned that the optimal cut-off to distinguish the no-responders from the responders on the early PET-CT scan was a reduction in tumour SUVmax of 61.2% (85.4% sensitivity, 65.2% specificity) [18].

An important issue remains unclear; that is, the PET-CT study method (timing, technique, and qualitative analysis of images). Most studies report inaccurate results due to heterogeneous methods for 18F-FDG PET-CT quantification, the correct time to perform the study and the metabolic criteria [19, 20]. It is important to standardize the criteria for the correct use of 18F-FDG PET-CT in order to achieve a correct interpretation of the results. In the current literature, there is no standardized data that indicates the proper timing by which to perform the 18F-FDG PET-CT. It is well known that chemotherapy produces an inflammatory reaction that lasts for one week after the beginning of treatment, while radiotherapy inflammatory reaction may last up to six months. Therefore, it is important choose or indicate the right time to perform 18F-FDG PET-CT after nCRT in order to standardize the correct interval as being a potential source of false-positive findings on the late 18F-FDG PET-CT. The World Health Organization recommends 18F-FDG PET-CT 7 weeks after nCRT and surgery one week later [28]. This is mainly based after the trial performed by R O Perez et al., showing a proper restaging with 18F-FDG PET-CT at 6 and 12 weeks after the completion of therapy [15]. The present study was concomitant with this recommendation, as all patients underwent FDG PET-CT 6–7 weeks after the end of nCRT, and surgery was performed 8 weeks from the end of neoadjuvant treatment.

The main limitations are the relatively small number of patients in our cohort due to a single centre enrolment. However, the unicentric nature of our study also represents a guarantee for more homogenous data.

We believe that more technically advanced tools are important to accurately measure tumour change. Nowadays the use of quantitative analysis of PET/MRI to assess pCR following nRCT in LARC could improve outcome predictions and may welcome the era of adaptive therapy for cancer patients [29, 30].

In conclusion, the results of this analysis are promising and show that 18F-FDG PET-CT may be an indicator to evaluate the pR to nCRT in patients with LARC. The decrease in 18F-FDG PET-CT uptake in the primary tumour may offer primary information for the early identification of patients who more likely to obtain a pCR to nCRT and to predict patients who are unlikely to significantly regress. Rigorous follow up and larger future prospective studies are necessary to confirm these results.
